# Mapping Mangrove Forests of Dongzhaigang Nature Reserve in China Using Landsat 8 and Radarsat-2 Polarimetric SAR Data

**DOI:** 10.3390/s18114012

**Published:** 2018-11-17

**Authors:** Jianing Zhen, Jingjuan Liao, Guozhuang Shen

**Affiliations:** 1 Key Laboratory of Digital Earth Science, Institute of Remote Sensing and Digital Earth, Chinese Academy of Sciences, Beijing 100094, China; zhenjn@radi.ac.cn (J.Z.); shengz@radi.ac.cn (G.S.); 2 Key Laboratory of Earth Observation Hainan Province, Sanya 572000, China; 3 University of Chinese Academy of Sciences, Beijing 100049, China

**Keywords:** mangrove forest, Radasat-2, Landsat 8, SVM, classification, mapping

## Abstract

Mangrove forests are distributed in intertidal regions that act as a “natural barrier” to the coast. They have enormous ecological, economic, and social value. However, the world’s mangrove forests are declining under immense pressure from anthropogenic and natural disturbances. Accurate information regarding mangrove forests is essential for their protection and restoration. The main objective of this study was to develop a method to improve the classification of mangrove forests using C-band quad-pol Synthetic Aperture Radar (SAR) data (Radarsat-2) and optical data (Landsat 8), and to analyze the spectral and backscattering signatures of mangrove forests. We used a support vector machine (SVM) classification method to classify the land use in Hainan Dongzhaigang National Nature Reserve (HDNNR). The results showed that the overall accuracy using only optical information was 83.5%. Classification accuracy was improved to a varying extent by the addition of different radar data. The highest overall accuracy was 95.0% based on a combination of SAR and optical data. The area of mangrove forest in the reserve was found to be 1981.7 ha, as determined from the group with the highest classification accuracy. Combining optical data with SAR data could improve the classification accuracy and be significant for mangrove forest conservation.

## 1. Introduction

Mangrove forests are swampy, woody plant communities that are distributed in the intertidal region between sea and land in tropical and subtropical coastlines. They have special sea-land characteristics and provide a “natural barrier” to the coast [1,2,3]. Mangrove forests have enormous ecological, economic, and social value [4,5,6,7,8,9]. They play an irreplaceable role in maintaining biodiversity, protecting the coastal environment, strengthening dykes, providing shelter from wind, protecting banks and inducing siltation, purifying the coastal water environment, and protecting farmland and villages from natural disasters, such as hurricanes and tsunamis. They are also indicators of global environmental and climate changes. However, the world’s mangrove forests are declining at an alarming rate, with 36% lost between 1995 and 2005, which is likely even more rapid than the loss of inland forests and tropical rainforests, and many of the remaining mangrove forests are in a degraded condition [10,11,12]. Due to the rapid development of the social and coastal economy, many mangrove forests have been converted into aquaculture and cultivated land [13,14]. The existing mangrove forests are under immense pressure from anthropogenic activities and natural disturbances. In the future, sea-level rise could be the biggest threat to mangrove ecosystems [15,16].

Mangrove forests are widely distributed in shallow mudflats between the sea and land. It is difficult to obtain accurate data by traditional field survey methods in such locations and they may even be completely inaccessible. Remote sensing, therefore, has significant advantages in the large-scale monitoring and analysis of mangrove forests and their environment, due to its merits of wide investigation range, abundant information, high efficiency, low cost, and few restrictions on ground conditions [17,18,19]. Remote sensing technology has been widely used in the classification and mapping of mangrove forests [20,21,22], dynamic monitoring [23,24], biomass estimation [25], ecological parameter estimation [18], and the impact of climate change and sea-level rise on mangrove forests [26].

Accurate information of mangrove forests is essential for determining the extent and distribution of mangroves, analyzing landscape change, identifying the rates and causes of changes in mangrove forests, and assessing the ecological health of mangroves [18,27,28,29]. Optical remote sensing, a primary method of extracting information regarding mangrove forests, records the difference between the spectral reflection and radiation characteristics of ground objects with a high spectral resolution and provides large amounts of information of the surface composition, enabling different objects to be identified [30,31]. However, optical remote sensing data are usually insufficient or missing due to mangrove forests being distributed in cloudy and rainy tropical and subtropical regions, which has encouraged the use of other remote sensing datasets, such as synthetic aperture radar (SAR) data. SAR data have many advantages, such as few limitations by time-weather conditions, side-looking imaging, high resolution, and good penetrability. It utilizes longer wavelengths than optical remote sensing and can therefore penetrate the vegetation canopy and better reflect the spatial structure of mangrove communities to obtain more accurate vegetation information [32]. SAR data have more advantages for estimating vegetation biomass than optical remote sensing [33,34]. Different bands (e.g., X-band, C-band, and L-band) have different backscattering signatures for the vegetation canopy, soil, and water surfaces, and radar remote sensing can be used to effectively monitor tree height, average crown width, health, and the degeneration of mangroves based on the backscatter coefficient [29,35,36,37,38]. Some studies had shown that both structural information and spectral signatures are required for improving the classification accuracy [39,40]. Liu et al used different fusion methods to fuse Radarsat-1 and Landsatdata for classifying mangrove communities in Qi’ao Island in Zhuhai. Zhang et al. used WorldView optical data and Radarsat-2 dual polarimetric SAR data to classify four mangrove species in the Mai Po Marshes Nature Reserve, Hong Kong, and improved the accuracy by 2–3% compared to results obtained by optical remote sensing data [41]. High-resolution optical data (Rapid Eye and WorldView-1) and L-band SAR data from the Advanced Land Observing Satellite/Phased Array type L-band Synthetic Aperture Radar (ALOS/PALSAR) was integrated to map the extent of mangroves along the Red Sea coastline in Egypt [20].

The main objective of this study was to develop a method to improve the classification of mangrove forests in Hainan Dongzhaigang National Nature Reserve (HDNNR) using C-band quad-pol polarimetric SAR data (Radarsat-2) and optical data (Landsat 8) and to analyze the spectral and backscattering signatures of mangrove forests. We used the support vector machine (SVM) classification method, which is a small sample learning machine that has the advantage of solving the nonlinear, high-dimensional pattern recognition, and small sample problems [42], to classify the land use in the HDNNR and its 2-km buffer zone. We also assessed the classification accuracy of different data categories and mapped the distribution of mangrove forests at a regional scale.

## 2. Materials and Methods

### 2.1. Study Area

**Figure 1:**
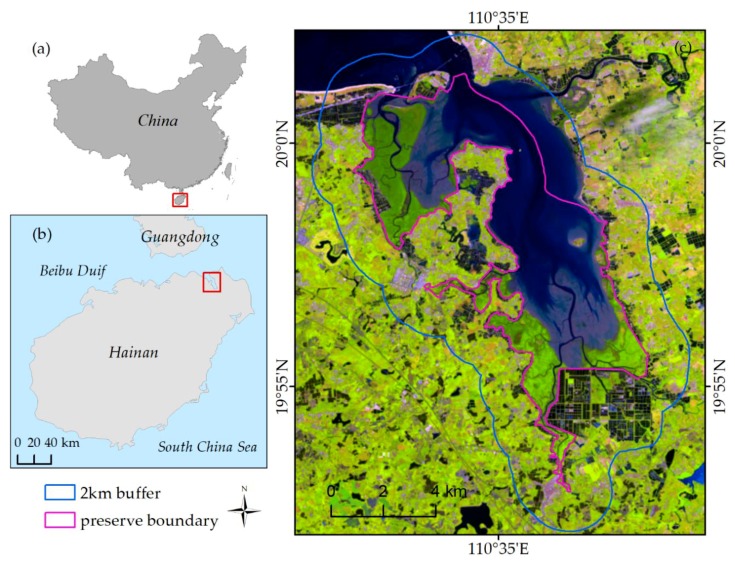
Location of the study area. (**a**): The location of Hainan province in China; (**b**): The location of study area in Hainan province; (**c**) The study area in Landsat 8 image (R: Shortwave infrared 1; G: Near Infrared; B: Red).

The HDNNR is the first mangrove wetland nature reserve in China and is located in the northeast of Hainan Island (between 110°32′E–110°37′E and 19°51′N–20°1′N), spanning the boundary between Haikou and Wenchang. It is the biggest and youngest bay in Hainan Island. It was formed by ground subsidence in a massive earthquake in 1605 and presents an irregular strip approximately in the south-north direction (long axis spanning 16 km, short axis spanning 8 km, and a width of 8 km at its widest point), with an area of 100 km^2^ (Figure 1). Silt and mud were deposited in the gentle and ladder-like tidal flat along the rugged coastline and funnel-shaped open bay. Mangrove forests and many zigzag-shaped tidal creeks are distributed in the bay, which is covered by sea water at high tide. An intersected tidal flat is exposed at low tide. 

The HDNNR is located at the northern edge of the tropics and has a tropical oceanic monsoon climate, which is characterized by humid warm springs, hot and rainy summers, heavy typhoon rainstorms in autumn, and cold wet winters. The annual average temperature is 23.3–23.8 °C, with an extreme high temperature of 38.9 °C in July and an extreme low temperature of 2.6 °C in January. The annual precipitation is 1676.4 mm, and the beginning and end of the rainy season are in early May and late October, respectively. The relative humidity is 85%, with a slight interannual variation resulting in a range of 82–88%. Dongzhaigang Bay is often referred to as “one harbor with four green rivers” with Yanzhou River to the east, Sanjiang River to the south, Yanfeng River to the east, and West River to the west. The four rivers and some short channels supply 0.7 billion m^3^ of water into Dongzhaigang Bay. In the rainy season, the rivers carry large quantities of sediment, which have deposited to form a flat swamp that is suitable for mangrove growth and propagation. The study area has an irregular semidiurnal tide, with a large tidal scope and a wide intertidal zone that provides plenty of growing space for mangrove forests. The dynamic force of the tide can propagate seeds for mangrove forests, supporting its growth and reproduction. Mudflats are the main soil type, and the soil parent materials are mainly basalts and olivine basalt in the bay, while a typical latosol has formed on the land under the zonal climate [43]. 

The mangrove forests in the HDNNR are the largest coastal tidal forests in China, and their excellent hydrothermal condition and the variety of habitats they provide encourage many aquatic animals (e.g., green crab, Penaeus monodon, and sandworm) to breed and forage in the area, particularly in the tree roots and tidal flats. The HDNNR has the best preserved and most concentrated, continuous, and mature mangrove forests and is the most resourceful among all mangrove-type nature reserves in China. With 25 true mangrove species (nine introduced), the reserve contains 96.2% of all mangrove species recorded in China [44]. It also has 40 species of semi-mangrove- and mangrove-associated species, generating high biodiversity levels. The tree species include Nypa fruticans, Lumnitzera littorea, Sonneratia hainanensis, Sonneratia ovata, Sonneratia paracaseolaris, Xylocarpus granatum, Rhizophora apiculate, and Acrostichum speciosum [45]. In this study, the HDNNR and its 2-km buffer zone, with an area of about 145 km^2^, were selected for study by analyzing remote sensing images and the reserve’s general condition (Figure 1).

### 2.2. Data 

#### 2.2.1. Remote Sensing Data

Optical remote sensing data and polarimetric SAR data were used to accurately map mangrove forest. (1) Optical remote sensing data: One Landsat 8 satellite image (path/row: 123/46) was acquired on 21 April 2017 and downloaded from the United States Geological Survey (USGS) website (http://www.usgs.gov). Landsat 8 ensures the continued acquisition and availability of Landsat data, and uses a two-sensor payload, the Operational Land Imager (OLI) and Thermal Infrared Sensor (TIRS). These two instruments collect image data for nine shortwave bands (panchromatic (PAN) images, with a high spatial resolution of 15 m and multispectral (MS) images with rich spectral information but a low spatial resolution of 30 m) and two longwave thermal bands. Additionally, two GF-2 images, with a central latitude and longitude (110.4°E and 19.9°N, 110.4°E and 20.1°N), were acquired on 9 December 2016 and downloaded from the Chinese High-Resolution Earth Observation System in Hainan Data and Application Center. The GF-2 satellite was designed by the Chinese High-Resolution Earth Observation System Major Project and carries two high-resolution cameras (PAN images with a resolution of 1 m and MS images with a resolution of 4 m). It is China’s first self-developed optical satellite, and has a spatial resolution of more than 1 m (Chinese Resources Satellite Application Center in Beijing, 2014). (2) Polarimetric SAR data: One Single Look Complex Radarsat-2 image, with HH (horizontal transmit and horizontal receive), VV, (vertical transmit and vertical receive) HV (horizontal transmit and vertical receive), and VH (vertical transmit and horizontal receive) polarization modes and operated in wide fine mode, was acquired over the study area on 18 May 2017. The data had azimuth and range resolutions of 4.78 and 4.73 m, respectively, and an incidence angle of 27.06°. The nominal pixel spacing of the Radarsat-2 data was about 8 m. Landsat 8 optical data and Radarsat-2 polarimetric SAR data were used to classify the land use in the study area and GF-2 high-resolution data were used to verify the classification accuracy. The details of the data used in this study are shown in Table 1.

#### 2.2.2. Field Data

**Figure 2:**
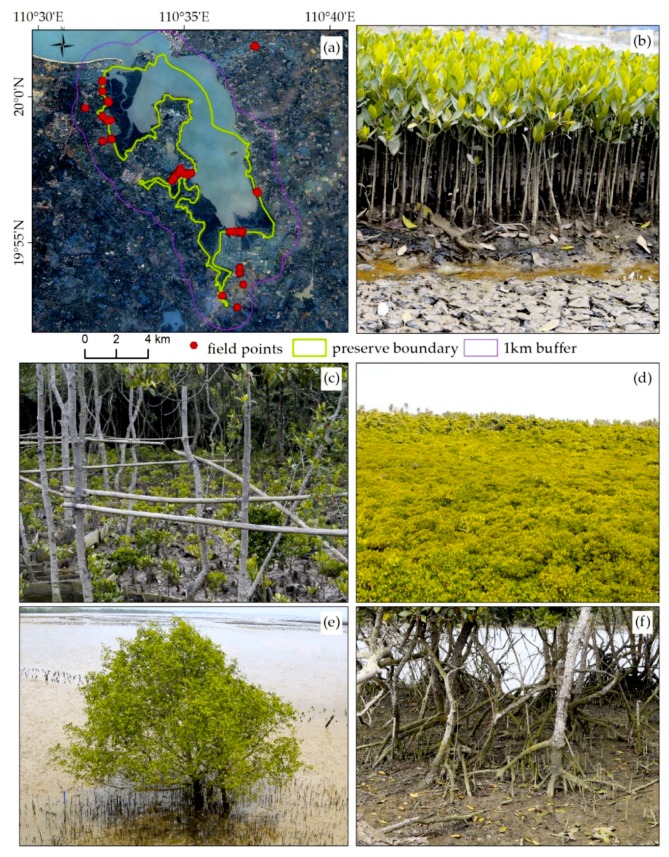
The verification points in the field. (**a**) The distribution of verification points overlaid on the GF-2 image; (**b**) seed cultivation of mangrove forests; (**c**) mangrove forest restored after a typhoon; (**d**) overlooking the mangrove forests from a wooden path in the reserve; (**e**) *Sonneratia caseolaris*; (**f**) *Kandelia candel*.

The field survey was carried out during the period of 17–25 March 2017 and the survey sites are shown in Figure 2. We used GPS, GF-2 images, and Google Earth images to collect 117 ground truth points, including 79 points of mangrove forests and 38 points of other land cover types and then investigated mangrove forest species, and their distribution, growth conditions, and surrounding environment. According to the survey results and those of previous studies, we defined nine classes in the study area (Table 2): mangrove forests (MF), building land (BDL), cultivated land (CL), other forest (OF), aquaculture ponds (AP), water (WT), bare land (BL), tidal sandflats (TS), and suitable land for mangrove (SLM). We selected 1863 samples (940 samples for training and 923 samples for validation) from GF-2 image referring to the field points and Google Earth images. The number of training samples and validation samples are shown in Table 2.

**Figure 3:**
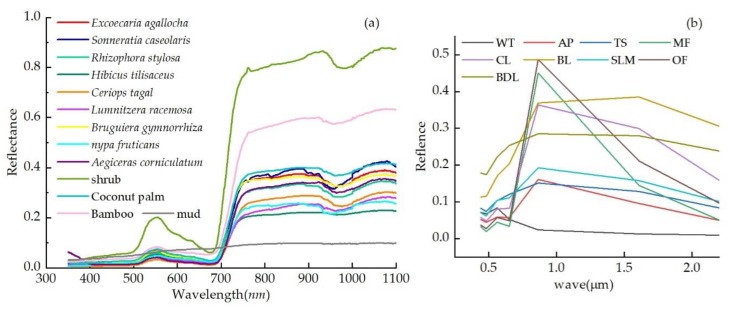
Spectral curves of mangrove forests and non-mangrove forests. (**a**) The spectral curves of different objects obtained using a field spectrometer; (**b**) the mean pixel value for each classification target in each multispectral (MS) band of a Landsat 8 satellite image. WT: water; AP: aquaculture pond; TS: tidal sandflats; MF: mangrove forests; CL: cultivated land; BL: bare land; SLM: suitable land for mangrove; OF: other forest; BDL: building land.

To analyze the spectrum characteristics of mangrove forests, we used an Analytical Spectral Devices (ASD) Field Spec Pro spectrometer to collect spectrum information by tilting the optical probe to about 45 degrees with respect to the crown of the mangrove forests. Measurements were taken under cloudless and windless conditions between 10:00 a.m. and 15:00 p.m. (Beijing local time). The spectrometer was operated in the spectral range of 350–2500nm and it has a sampling interval of 1.4 nm between 350 and 1050 nm and 2 nm between 1050 and 2500 nm. The spectral resolution is 3 nm at 700 nm and 10 nm at 1400 nm. We used a 40 × 40 cm diffuse white calibration panel made by BaSO_4_ to calculate the baseline reflectance with the optimal illumination condition. Vegetation radiance measurements were taken by averaging 15 scans at an optimized integration time. A panel radiance measurement was taken before and after the vegetation measurement by two scans each time. Ninety-seven field spectral samples were collected, including samples of *Sonneratia caseolaris*, *Bruguiera gymnoihiza*, *Ceriops tagal*, *Lumnitzera racemose*, *Aegiceras corniculatum*, shrubs, bamboo, coconut palm, and mud. Their reflectance values were exported using ViewSpecPro software, large errors were removed, and the spectral curve was averaged using MATLAB software. The spectral curves of mangrove forests and non-mangrove forests are shown in Figure 3a.

Mangrove forests have the typical spectral response features of green plants, as shown in Figure 3a. There are two absorbing regions in the blue and red bands, with center wavelengths of 450 and 670 nm, respectively, whereas they form a green reflection peak at the center wavelength of 540 nm. A “red edge” exists from 675 to 750 nm, with the reflection increasing from the red to near-infrared wavelengths. In the near-infrared wavelength (740–1100 nm), the spectral features of mangrove forests depend on the inner structures of leaves. The difference in the refractive index between cell walls and the gaps in leaves forms multiple reflections, resulting in a high reflectance.

It is easy to distinguish between mangrove forests and mud because there is an obvious difference between their spectral curves. Mangrove forests, bamboo, and coconut palm are all green plants, and their spectral curves display the same curve trend. Due to the special habitats of mangrove forests, their underlying surface has a larger heat absorption and lower reflectance than shrubs, bamboo, and coconut palm [34], and therefore the reflectance of terrestrial vegetation is significantly higher than that of mangrove forests at 700–1100 nm. Ten typical samples of Landsat 8 image were selected for each class to analyze their spectral features and are shown in Figure 3b. The spectral curve of mangrove forests displayed the same trends as other vegetation types (other forests and cultivated land) in both the visible and near-infrared wavelengths and a lower reflectance in the short infrared wavelength. Due to the periodic inundation of mangrove forest habitats, the spectral features of mangrove forests at short infrared wavelengths are similar to those of vegetation-water-mixed pixels and their reflectance is different from terrestrial vegetation in satellite images [46]. 

### 2.3. Methods

#### 2.3.1. Image Pre-Processing 

The ENVI 5.3 software was used to pre-process Landsat 8 and GF-2 images. Radiometric calibration, atmospheric correction, and image fusion and study area subset were conducted for the Landsat 8 image. Additionally, the normalized difference vegetation index (NDVI) was calculated using the red and near-infrared bands. Orthorectification, radiometric calibration, atmospheric correction, geometric rectification, and image fusion and study area subset were conducted for the GF-2 image. Atmospheric correction was conducted using the FLAASH module of ENVI 5.3 [47]. The Gram-Schmidt fusion method was used to fuse Landsat 8 and GF-2 images to 15 and 1 m, respectively. This is a high-fidelity fusion method and can maintain the consistency of image spectral information before and after fusion [48].

The SNAP software, which is provided by the European Space Agency (ESA), was used to pre-process Radarsat-2 images, including radiometric correction, polarization filter, multi-look, polarization decomposition, and terrain correction. After radiometric correction, the data were transformed to dB units. For the purpose of this analysis, it was assumed that HV was approximately equal to VH, which is typically the case for most natural targets [49,50,51]. To suppress the uncertainty due to speckle noise in the Radarsat-2 image, the Refined Lee Filter was applied using a 5 × 5 pixel window [52], which avoids the crosstalk between polarimetric channels as well as maintaining the polarization information and statistical correlations. Multi-look techniques were also conducted, with one look in the azimuthal direction and two looks in the range direction to obtain a resolution of 9.8 m in the Radarsat-2 image. The Freeman-Durden [53] and Yamaguchi [54] decompositions were then conducted. To add geographical information and correct the geometric distortion in the image, a Range Doppler Terrain Correction was conducted using the Shuttle Radar Topography Mission (SRTM) 30 m digital elevation model (DEM). Other SAR variables were calculated, including the HH-VV difference, HV-HH difference, and HH/HV intensity ratios.

**Figure 4:**
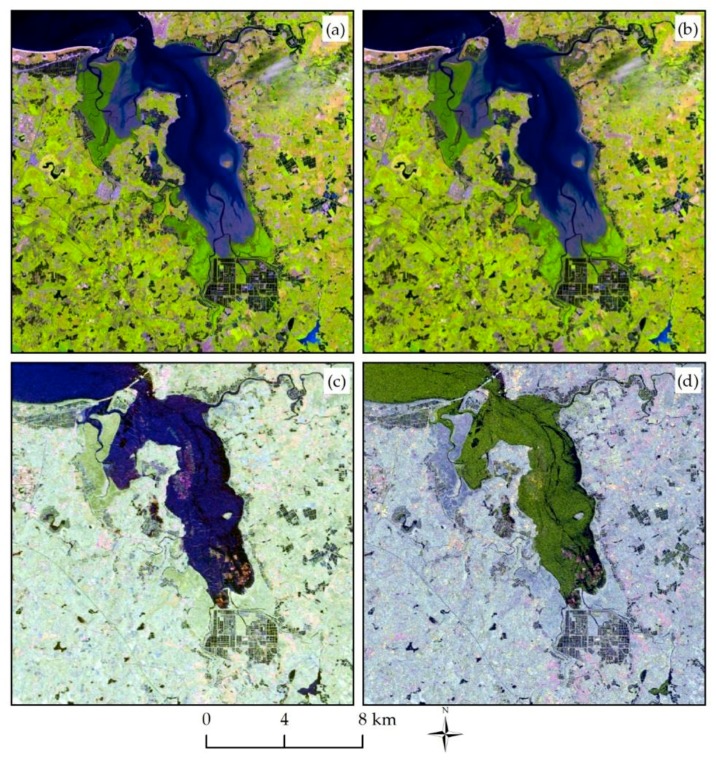
Different remote sensing data for the study area: (**a**) Landsat 8 image (R: 6, G: 5, and B: 4); (**b**) the fused images between panchromatic (PAN) and MS images of Landsat 8; (**c**) Pauli decomposition of the Radarsat-2 image; and (**d**) false-color synthetic image of the Radarsat-2 polarimetric channels (R: HH, G: VV, and B: HV).

We stacked all Radarsat-2 SAR layers (HH, HV, VV, HH-VV, HV-HH, HH/HV, Freeman_dbl, Freeman_vol, Freeman_surf, Yamaguchi_dbl, Yamaguchi_vol, Yamaguchi_surf, and Yamaguchi_hlx) and Landsat 8 layers (seven MS bands after image fusion and application of the NDVI) separately to obtain SAR datasets and optical datasets using the Layer Stacking Tool of the ENVI 5.3 software. Then, the SAR data were resampled to 15 m and made registration with an optical dataset, with an error of less than 0.5 pixels. Finally, the two datasets were combined to map mangrove forests using different variables. Different remote sensing data for the study area are shown in Figure 4. 

**Figure 5:**
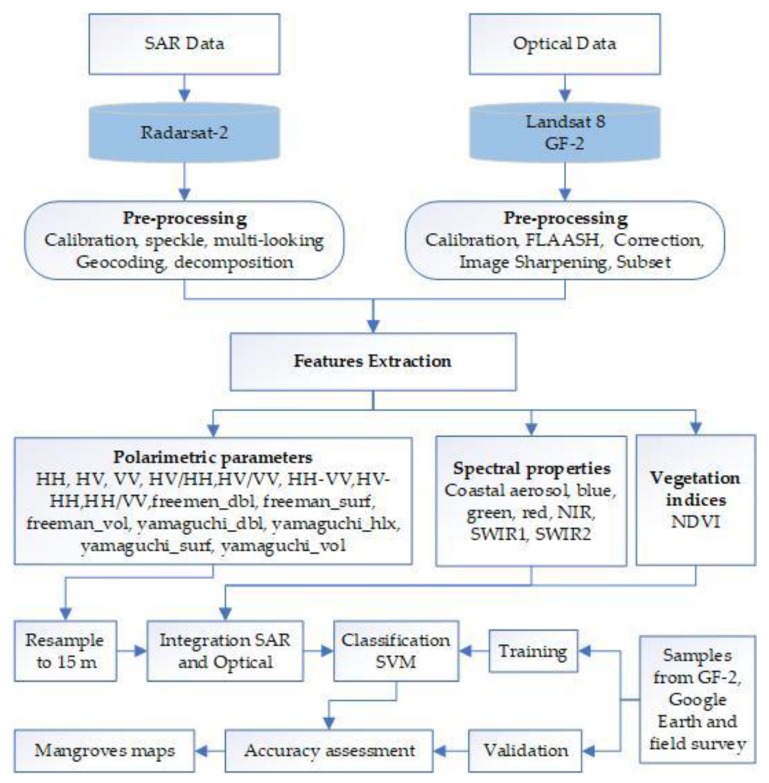
Flowchart of the proposed methodology.

The methodological framework used in this study is shown in Figure 5.

#### 2.3.2. Image Classification

The SVM method is a machine learning method based on Vapnik–Chervonenkis Dimension theory and the structural risk minimizing principle [55]. It attempts to locate an optimal hyperplane that maximizes the margin between two classes in high-dimensional space, and has been applied in remote sensing image classification [56,57,58]. For two linearly separable samples, this classifier classifies data by finding an optimal hyperplane that separates all of the data points of one class from those of another class. The hyperplane only needs a few samples to be determined and constructs support vectors. For two non-linearly separable samples, the classifier maps the vector from low- to high-dimensional space using a kernel function [59]. The SVM has good general applicability and can transform non-linear problems to linear problems by constructing a discrimination function in high-dimensional space; therefore, it is not influenced by sample dimensions and can avoid dimensional disaster [60]. A radial basis function (RBF) was applied in this study, with the penalty factor set at 100 and Gamma function set at 0.022. This kernel function maps a single vector to a vector of higher dimensionality and has good performance regardless of the sample size.

Three classification scenarios were applied in this study. The first scenario (OD—optical data) used only optical image information, with seven Landsat 8 MS bands (Coastal, Blue, Green, Red, Near Infrared Shortwave Infrared1, and Shortwave Infrared2) and the calculated NDVI value. The second scenario (SD—SAR data) used all the SAR information, with three Radarsat-2 SAR bands (HH, HV, and VV), polarimetric decomposition parameters (Freeman_dbl, Freeman_vol, Freeman_surf, Yamaguchi_dbl, Yamaguchi_vol, Yamaguchi _surf, and Yamaguchi_hlx), and other SAR variables (HH-VV, HV-HH, and HH/HV). The third scenario (IOSD—integrated optical and SAR data) combined both optical image information and SAR information to examine the potential for mapping the extent of mangrove forests. Table 3 shows the details of each category for the three scenarios.

#### 2.3.3. Accuracy Assessment

Accuracy was assessed by comparing the real surface data with the classification results and is an essential part of remote sensing image classification. The Kappa coefficient and a confusion matrix are usually used for the accuracy assessment of classified images in a remote sensing image classification accuracy assessment system. In this study, accuracy assessment was carried out for the classification images using high-spatial-resolution GF-2 image. Firstly, we stacked the 923 validation samples selected from GF-2 image before the combined datasets to check the validation samples and make adjustment if necessary. Then, we used the Confusion Matrix using Ground Truth ROIs tool of ENVI5.3 software to assess the classification results. Finally, the parameters of overall accuracy, producer accuracy, user accuracy, and the Kappa coefficient were derived from the confusion matrix and used for an accuracy assessment of the classified images (Table 4).

## 3. Results and Discussion

### 3.1. Analysis of the Backscattering Characterization and Polarimetric Decomposition 

**Figure 6:**
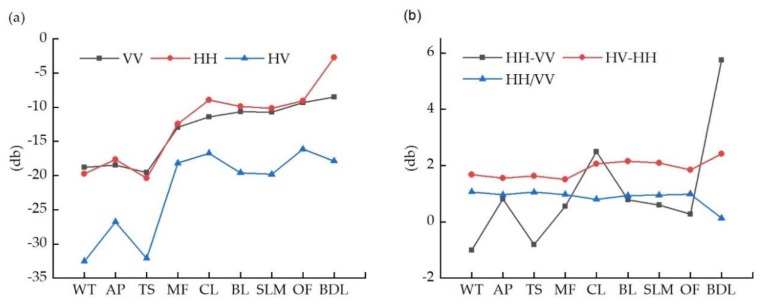
(**a**) Mean backscattered values of each land use class in the three channels (HH, HV, and VV); (**b**) values of the principal components in the three channels (HH, HV, and VV). WT: water; AP: aquaculture pond; TS: tidal sandflats; MF: mangrove forests; CL: cultivated land; BL: bare land; SLM: suitable land for mangrove; OF: other forest; BDL: building land.

The mean backscattering coefficients of the different polarimetric channels (HH, VV, and HV) of the nine land cover types in the study area were extracted, i.e., MF, BDL, CL, OF, AP, WT, BL, TS, and SLM. Table 4 and Figure 6a show the variation of the backscattering coefficients for each class at different polarizations of HH, VV, and HV. The backscattering intensity was lowest for WT, with values in the ranges of −25.56 to −10.33, −36.97 to −15.57, and −28.62 to −11.99 dB in the HH, VV, and HV channels, respectively. This was because the radar antenna could not receive echoes when the smooth water surface produces specular reflections. The backscattering intensity of TS was almost as low as that of WT due to the specular scattering of microwave radiation by the overlaying water [61]. The mean backscattering coefficients of MF in the HH, VV, and HV channels were −12.41, −18.18, and −12.96 dB, respectively. Due to the depolarization of MF, its HV backscattering coefficient was lower than its HH and VV backscattering coefficients. Due to its distribution in shallow mudflats between the sea and land, the special underlying surface of MF resulted in the backscattering coefficients in the three channels being lower than those for OF and CL, and distinguished MF from the other classes. CL and BL had high backscattered coefficients due to their rough surface, resulting in the tensive scattering of electromagnetic waves. The highest backscattered coefficients were observed for BL due to its special geometry and surface materials, with a large dielectric constant. SMF had a high soil water content and dielectric constant and also had high backscattering values, especially in the HH polarization channel.

**Figure 7:**
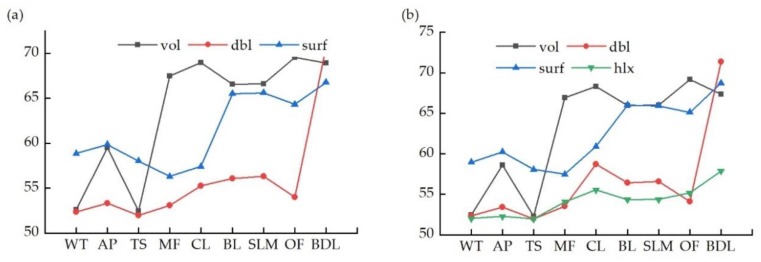
(**a**) The three components (surface scattering, double scattering, and volumetric scattering) of the Freeman polarimetric decomposition for each class; (**b**) the three components of the Yamaguchi polarimetric decomposition for each land use class. WT: water; AP: aquaculture pond; TS: tidal sandflats; MF: mangrove forests; CL: cultivated land; BL: bare land; SLM: suitable land for mangrove; OF: other forest; BDL: building land.

The target scattering matrix obtained from polarimetric SAR data usually reflects the mean scattering characteristics of the scattering target. However, polarimetric SAR target decomposition decomposes the complex scattering process of a surface echo to several single scattering matrices that are helpful for analyzing radar target scattering properties and interpreting the scattering mechanism of ground objects. Figure 7 shows the mean power of the three scatterings (surface scattering, double scattering, and volumetric scattering) for the Freeman and Yamaguchi decompositions of the training samples of each class on the Radarsat-2 image. MF, CL, and OF were all vegetation types and therefore volumetric scattering was the most useful approach, with OF having the highest value. MF had the lowest surface scattering and double scattering values and could be easily distinguished from CL and OF. The double scattering of BDL was highest in all classes, and was followed by TS. Surface scattering was the most useful approach for WT, which had low double scattering and volumetric scattering values, while AP had high surface scattering and volumetric scattering values and lower double scattering values. BL and SLM also had high surface scattering and volumetric scattering values and lower double scattering values due to reflectance from the bare surfaces.

### 3.2. Analysis of the Classification Results 

**Figure 8:**
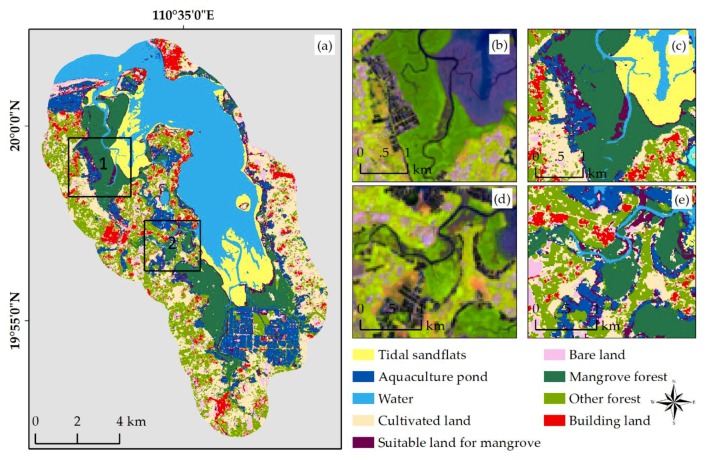
Classification results. (**a**) Classification results in category IOSD5; (**b**,**c**) show the Landsat 8 image and the classification results in position 1 of (**a**); (**d**,**e**) show the Landsat 8 image and classification results in position 2 of (**a**). WT: water; AP: aquaculture pond; TS: tidal sandflats; MF: mangrove forests; CL: cultivated land; BL: bare land; SLM: suitable land for mangrove; OF: other forest; BDL: building land.

**Figure 9:**
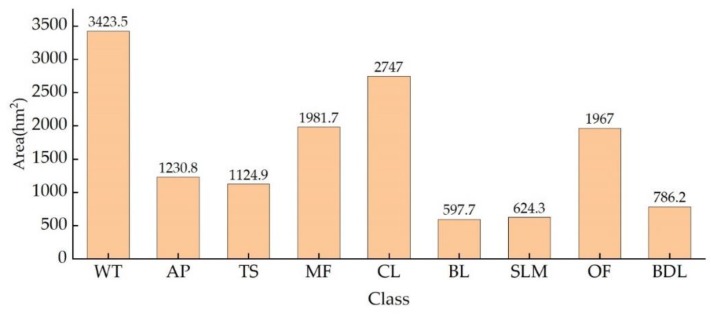
The area of each land use class in the study area. WT: water; AP: aquaculture pond; TS: tidal sandflats; MF: mangrove forests; CL: cultivated land; BL: bare land; SLM: suitable land for mangrove; OF: other forest; BDL: building land.

An accuracy assessment was conducted for each classified image for the three classification scenarios. Quantitative evaluation indices (overall accuracy, producer accuracy, user accuracy, and the Kappa coefficient) were obtained according to the confusion matrix (Table 5). The overall accuracy was 83.5% and 53.4% using only optical information and SAR information, respectively. However, when using both optical information and SAR information, the overall user accuracy (IOSD5) of MF was greater than in the other two scenarios at 95.0% and 96.7%. Figure 8 shows the classification results of IOSD5. The area of each class (WT, CP, TS, MF, CL, BL, SLM, OF, and BDL) in the study area was 3423.5, 1230.8, 1124.9, 1981.7, 2747, 597.7, 624.3, 1967.0, and 786.2 ha (Figure 9), respectively. The Dongzhaigang coastline is dominated by strips of MF, while CL, OF, and AP also account for a large proportion of the land area. The other classes are embedded among these land uses. MF, including both open and closed forests, account for 13.68% of the land area, while AP account for 8.50%. MF are distributed in areas with a fragile ecology and are degrading throughout the reserve due to human activities [62,63]. Many natural and human factors currently influence the ecosystems of this reserve, including typhoons, tides, the discharge of pollutants, reclamation of tidal flats, cultivation, and overfishing, which exert great pressure on MF [64,65]. Wang et al. conducted an investigation on the mangrove community in 2012 and 2014 and found the ability of mangrove roots to fix soil had weakened due to an outbreak of Sphaeroma in the reserve in 2010 [63]. Mangrove degradation was further aggravated by the influence of typhoon “Rammasun” in July 2014 [66].

Table 5 provides a summary of the classification results obtained from all data categories. In category OD, the overall accuracy of subgroup OD1 was 83.5%, with a Kappa coefficient of 0.80. After adding the NDVI, the classification accuracy improved slightly to 84.1%, with a Kappa coefficient of 0.81. There was no significant increase in overall accuracy, but the accuracy of MF and BL were improved to some extent. The NDVI is the best indicator of vegetation growth status and coverage, and can effectively distinguish between areas with or without vegetation cover.

In category SD, the overall accuracy of each subgroup was 53.4%, 53.5%, 59.6%, and 63.9%, respectively. The addition of polarimetric decomposition parameters and other SAR variables to the three SAR polarizations of Radarsat-2 improved the classification accuracy, while the accuracy was relatively low when using SAR data only.

In category IOSD (combined optical and SAR data), the overall accuracy of subgroups IOSD1–IOSD5 was between 88.95% and 95.04%, which was higher than for categories OD and SD. In addition, the user accuracies of MF, CL, and OF were significantly improved. From the analysis in Section 3.1, it was found that the different scattering signatures of different objects in each SAR channel increased the distinguishability, and improved the classification accuracy in subgroup OISD1. In subgroup IOSD2 (seven MS bands and HH-VV, HV-HH, and HH/VV), the user accuracy of MF, and the producer accuracies of BDL and CL were all improved to a certain extent. HH polarization mainly reflects the backscattering capability of an objects’ surface, while HV polarization is sensitive to volume scattering, and HH/VV reflects the depolarizing ability. BDL has the highest value at HV-HH and lowest value at HH/VV, while other land cover classes were similar in these two channels. In the HH-VV channel, BDL had the highest value and WT had the lowest, while MF were similar to the other land cover classes. Polarimetric decomposition parameters (Freeman and Yamaguchi decompositions) were added to subgroups IOSD3 and IOSD4 and the overall accuracies were improved to 93.07% and 92.73%, respectively, which were higher than in category OD.

The highest overall classification accuracy was obtained from subgroup GC5, with an overall accuracy of 95.04% and a Kappa coefficient of 0.94 based on a combination of SAR backscattering, polarimetric decomposition parameters, and other SAR variables with surface reflectance of the optical data. The results indicate that the combination of SAR and optical data can increase the separability between features, and improve the classification accuracy to some extent.

## 4. Conclusions

Accurate classification and mapping of mangrove forests is crucial. In this study, we used optical remote sensing data from Landsat 8 and full-polarization C-band SAR data from Radarsat-2 to map the extent of mangrove forests in the HDNNR by applying an SVM classifier with high dimensionality and the capability of solving non-linear problems. The classification results for different datasets were assessed through the overall accuracy, producer accuracy, user accuracy, and Kappa coefficient.

Mangrove forests, with the typical spectral response features of green plants, could be easily distinguished from water, bare land, and built-up land. However, the spectral curve of mangrove forests displayed the same trend as other vegetation types (other forest types and cultivated land) in both the visible and near-infrared wavelengths, but there was a certain distinguishability in the short infrared wavelength. Optical remote sensing could obtain better classification results due to its high resolution and strong interpretation. Although more accurate spatial information for vegetation can be obtained by radar remote sensing due to its strong penetration and useful polarization information, the classification accuracy is not ideal when using SAR data only. In this study, the overall accuracy ranged from 83.5% using only optical data to 95.04% when combining optical and SAR data. Different variables (NDVI, HH-VV difference, HV-HH difference, HH/HV intensity ratios and polarization decomposition parameters) derived from optical and SAR data had improved the accuracy to some extent. 

This indicates that multi-dimensional features from different remote sensing images can be used to map the mangrove forests extend by the SVM machine learning method. SAR data not only compensate the shortcomings of optical remote sensing, but also are useful for improving the classification accuracy and mapping the extent of mangrove forests when it is combined with optical data. Furthermore, optical image with high spatial resolution, combined with SAR image with high spatial resolution and longer wavelength, will achieve mangrove forests species discrimination. This combination will provide more accurate information, enabling the study of the spatial distribution and dynamic changes of mangrove forests, which is important for establishing conservation and management policies. 

## Figures and Tables

**Table 1:** Details of the remote sensing data.

Satellite	Acquisition Date	Spectral/Polarizations		Resolution
Landsat 8	21 April 2017	Pan	0.500–0.680 μm	15 m
		Coastal	0.433–0.453 μm	30 m
		Blue	0.450–0.515 μm	
		Green	0.525–0.600 μm	
		Red	0.630–0.680 μm	
		NIR	0.845–0.885 μm	
		SWIR1	1.560–1.660 μm	
		SWIR2	2.100–2.300 μm	
GF-2	9 December 2016	Pan	0.45–0.90 μm	1 m
		Blue	0.45–0.52 μm	4 m
		Green	0.52-0.59 μm	
		Red	0.63–0.69 μm	
		NIR	0.77–0.89 μm	
Radarsat-2	18 May 2017	HH, HV, VH, VV		8 m

**Table 2:** Definitions of the classes used in this study.

Classes	Definition of Support Vector Machine (SVM) Classification	Training Samples	Validation Samples
Mangrove forests (MF)	Tidal marsh covered by both closed and open mangrove forests	177	152
Building land (BDL)	Rural residential land, urban construction land, and industrial and mining areas	80	104
Cultivated land (CL)	Land covered by crops	195	176
Other forest (OF)	Land covered by forest other than mangrove forests	89	113
Aquaculture ponds (AP)	Mainly distributed between the coastline and cultivated land or forests, e.g., fish ponds, and shrimp ponds	68	65
Water (WT)	Areas of open water with no emergent vegetation	105	84
Bare land (BL)	Areas devoid of vegetation	93	91
Tidal sandflats (TS)	Loose beach consisting of sand or gravel with little vegetation cover	68	72
Suitable land for mangrove (SLM)	Coastal or riparian wetland suitable for mangrove forests	65	66

**Table 3:** Feature vector selection of the three schemes used for classification.

Scenario		Selected Features and Combinations
OD	OD1	Coastal, Blue, Green, Red, NIR, SWIR1, SWIR2
	OD2	Coastal, Blue, Green, Red, NIR, SWIR1, SWIR2, NDVI
SD	SD1	HH, HV, VV
	SD2	HH, HV, VV, HH-VV, HV-HH, HH/HV
	SD3	HH, HV, VV, HV/HH, VV/HH, Freeman_dbl, Freeman_vol, Freeman_surf
	SD4	HH, HV, VV, HV/HH, VV/HH, RPC1, RPC2, RPC3, Freeman_dbl, Freeman_vol, Freeman_surf
IOSD	IOSD1	Coastal, Blue, Green, Red, NIR, SWIR1, SWIR2, HH, HV, VV
	IOSD2	Coastal, Blue, Green, Red, NIR, SWIR1, SWIR2, HH-VV, HV-HH, HH/HV
	IOSD3	Coastal, Blue, Green, Red, NIR, SWIR1, SWIR2, Freeman_dbl, Freeman_vol, Freeman_surf
	IOSD4	Coastal, Blue, Green, Red, NIR, SWIR1, SWIR2, Yamaguchi_dbl, Yamaguchi_vol, Yamaguchi_surf, Yamaguchi_hlx
	IOSD5	Coastal, Blue, Green, Red, NIR, SWIR1, SWIR2, NDVI, HH, HV, VV, HH-VV, HV-HH, HH/HVFreeman_dbl, Freeman_vol, Freeman_surf

OD: optical data; SD: SAR data; IOSD: integrated optical and SAR data; NIR: near-infrared; NDVI: normalized difference vegetation index.

**Table 4:** Backscatter statistics for Radarsat-2 data for each class.

Class	HH Backscattering (dB)				HV Backscattering (dB)				VV Backscattering (dB)			
	Min	Max	Mean	SD	Min	Max	Mean	SD	Min	Max	Mean	SD
WT	−25.56	−10.33	−19.55	2.17	−36.97	−15.57	−32.47	2.55	−28.62	−11.99	−18.42	2.38
AP	−27.43	−7.78	−20.35	3.04	−36.28	−20.48	−32.12	1.82	−29.01	−7.78	−19.54	3.17
TS	−24.64	−4.82	−14.30	3.03	−34.45	−19.05	−27.51	2.98	−24.06	−7.78	−16.21	3.12
MF	−21.78	−5.25	−12.41	2.56	−25.97	−12.44	−18.18	2.21	−22.47	−5.60	−12.96	2.79
CL	−20.05	−1.91	−8.94	2.55	−27.34	−11.47	−16.74	1.68	−19.64	−4.46	−11.43	2.16
BL	−21.70	−2.99	−9.88	3.19	−31.37	−12.94	−19.60	3.44	−21.51	−2.50	−10.67	3.23
SLM	−20.37	−2.95	−10.12	3.32	−33.93	−13.03	−19.81	3.61	−21.86	−4.51	−10.71	3.03
OF	−14.70	−3.90	−9.04	1.58	−20.89	−11.79	−16.14	1.38	−15.83	−4.23	−9.31	1.65
BDL	−12.01	15.84	−2.74	5.30	−24.97	−8.80	−17.89	2.56	−15.84	11.18	−8.49	3.00

**Table 5:** Classification results of the three categories for each group. WT: water; AP: aquaculture pond; TS: tidal sandflats; MF: mangrove forests; CL: cultivated land; BL: bare land; SLM: suitable land for mangrove; OF: other forest; BDL: building land.

Group		OA (%)	Kappa (%)	MF		BDL		OF		WT		AP		CL		TS		SLM		BL	
				PA (%)	UA (%)	PA (%)	UA (%)	PA (%)	UA (%)	PA (%)	UA (%)	PA (%)	UA (%)	PA (%)	UA (%)	PA (%)	UA (%)	PA (%)	UA (%)	PA (%)	UA (%)
OD	OD1	83.5	0.80	90.3	90.6	90.3	90.2	67.4	83.9	93.2	97.2	88.4	86.4	86.0	64.8	87.4	87.3	55.5	74.1	73.0	73.4
	OD2	84.1	0.81	91.9	92.7	90.8	89.5	67.9	83.8	93.4	97.1	88.8	84.6	86.6	65.5	87.6	87.8	55.8	75.7	76.3	77.3
SD	SD1	53.4	0.46	55.21	57.63	40.45	55.34	72.31	41.73	95.59	63.82	73.81	60.4	49.54	49.96	17.63	63.64	4.36	36.11	14.42	42.62
	SD2	53.5	0.46	56.5	57.7	40.3	54.9	71.8	42.1	95.8	63.7	73.6	60.0	49.3	50.6	17.5	64.1	7.1	30.9	13.3	41.9
	SD3	59.6	0.53	60.5	67.1	55.0	68.9	79.6	46.4	94.3	64.6	83.9	73.4	49.9	58.6	28.7	83.6	16.8	20.8	28.3	62.2
	SD4	63.9	0.58	70.1	67.9	65.3	90.3	79.2	50.0	92.9	65.7	86.3	74.0	55.7	64.6	33.0	86.4	16.1	28.9	37.3	58.4
IOSD	IOSD1	88.95	0.87	87.1	95.5	96.9	94.2	79.8	89.7	95.9	94.1	90.7	88.2	91.8	79.4	90.8	90.7	83.9	84.2	79.9	93.5
	IOSD2	91.66	0.90	90.5	96.3	96.9	94.6	81.9	89.6	97.4	96.4	94.3	88.8	92.4	84.0	95.6	96.4	89.3	93.3	87.4	94.2
	IOSD3	93.07	0.92	94.3	95.5	95.9	98.5	84.9	92.5	96.3	93.4	93.6	92.2	94.4	88.8	93.8	96.5	89.9	93.1	93.7	93.0
	IOSD4	92.73	0.92	93.8	96.8	95.9	98.9	83.3	90.5	95.9	94.0	95.1	90.8	93.7	87.3	93.0	96.5	92.3	93.2	93.9	94.3
	IOSD5	95.04	0.94	94.2	96.7	95.5	97.7	91.2	93.7	97.4	98.7	97.2	92.3	95.2	92.6	98.8	98.8	90.9	91.3	93.7	92.7
